# West African Genetic Ancestry, Neighborhood Deprivation, and Prostate Cancer

**DOI:** 10.1001/jamanetworkopen.2024.33546

**Published:** 2024-09-16

**Authors:** Catherine M. Pichardo, Adaora Ezeani, Amanda M. Acevedo, Tanya Agurs-Collins, Maeve Bailey-Whyte, Tiffany H. Dorsey, Alexandra R. Harris, Jamirra Franklin, Rick A. Kittles, Wayne R. Lawrence, Christopher A. Loffredo, Tsion Zewdu Minas, Margaret S. Pichardo, Brid M. Ryan, Wei Tang, William Wooten, Jia Liu, Stefan Ambs

**Affiliations:** 1 Division of Cancer Control and Population Sciences, National Cancer Institute, National Institutes of Health, Rockville, Maryland; 2 Laboratory of Human Carcinogenesis, National Cancer Institute, National Institutes of Health, Bethesda, Maryland; 3 School of Medicine, University of Limerick, Limerick, Ireland; 4 Department of Community Health and Preventive Medicine, Morehouse School of Medicine, Atlanta, Georgia; 5 Division of Cancer Epidemiology & Genetics, National Cancer Institute, National Institutes of Health, Rockville, Maryland; 6 Cancer Prevention and Control Program, Lombardi Comprehensive Cancer Center, Georgetown University Medical Center, Washington, District of Columbia; 7 Department of Surgery, Hospital of the University of Pennsylvania, Penn Medicine, Philadelphia; 8 Data Science and Artificial Intelligence, Research and Development, AstraZeneca, Gaithersburg, Maryland; 9 University of Maryland Marlene and Stewart Greenebaum Comprehensive Cancer Center Biostatistics Shared Service, Baltimore; 10 Cancer Genomics Research Laboratory, National Cancer Institute, Rockville, Maryland

## Abstract

**Importance:**

Racial disparities in prostate cancer are likely the result of complex relationships between both socioeconomic and environmental factors captured by the neighborhood environment and genetic factors, including West African genetic ancestry. However, few studies have examined the combined role of neighborhood environment and genetic ancestry in developing lethal prostate cancer.

**Objective:**

To examine the interactions between West African genetic ancestry and neighborhood deprivation in modifying prostate cancer risk and mortality.

**Design, Setting, and Participants:**

This case-control study was conducted in the Greater Baltimore area. Participants included men of African and European descent (617 cases with prostate cancer, 852 controls without prostate cancer) enrolled between January 2005 and January 2016. Follow-up was performed through December 31, 2020, using the National Death Index. Analysis was conducted from August 2023 to January 2024.

**Exposure:**

Included exposures were West African genetic ancestry, derived from large-scale genotyping, and neighborhood deprivation, defined using 2000 census-tract–level Neighborhood Deprivation Index (NDI) score.

**Main Outcomes and Measures:**

Outcomes of interest were prostate cancer and all-cause mortality.

**Results:**

Among a total of 1469 participants (mean [SD] age, 64.96 [7.95] years), there were 733 self-identified Black and 736 White men, and the mean (range) proportion of West African genetic ancestry was 0.27 (0.04-0.84) among participants residing in areas with low levels of deprivation and 0.48 (0.07-0.83) among participants residing in areas with high levels of deprivation. Multivariable logistic regression analysis revealed a significant multiplicative interaction of West African genetic ancestry and neighborhood deprivation with the odds of a prostate cancer diagnosis (*P* for interaction = .02). Among individuals living in neighborhoods with high NDI scores, West African genetic ancestry was associated with increased odds of a prostate cancer diagnosis (age-adjusted odds ratio [OR], 1.98; 95% CI, 1.23-3.19). In contrast, West African genetic ancestry was associated with reduced odds of this diagnosis among individuals residing in areas with medium to low levels of deprivation (age-adjusted OR, 0.22; 95% CI, 0.11-0.44). There was no significant multiplicative interaction between West African genetic ancestry and neighborhood deprivation for all-cause mortality (*P* for interaction = .44). The positive association of neighborhood deprivation with prostate cancer was independent of West African genetic ancestry (age- and West African ancestry–adjusted OR, 1,70; 95% CI, 1.50-1.94).

**Conclusions and Relevance:**

This case-control study of men with West African and European ancestry found that West African genetic ancestry was associated with increased odds of prostate cancer among males who resided in neighborhoods with high deprivation but lower odds in more affluent neighborhoods. Thus, neighborhood environments may play a critical role in defining how genetic ancestry modulates prostate cancer risk.

## Key Points

**Question:**

Is genetic ancestry associated with prostate cancer risk and all-cause mortality in the context of neighborhood deprivation?

**Findings:**

In this case-control study of 1469 participants, West African genetic ancestry was associated with increased odds of a prostate cancer diagnosis among individuals living in neighborhoods with high levels of deprivation but lower odds of diagnosis among individuals living in neighborhoods with low deprivation. There was no association with all-cause mortality among men with prostate cancer.

**Meaning:**

The findings of this study suggest that neighborhood environments may influence the association of genetic ancestry with prostate cancer risk.

## Teaser

This case-control study examines the interactions between West African genetic ancestry and neighborhood deprivation in association with modifying prostate cancer risk and mortality among Black and White US men.

## Introduction

The incidence of prostate cancer is more than 70% higher in Black men than White men in the US.^1^ The disparity in prostate cancer mortality is similarly pronounced, and rates are more than twice as high among Black men.^1^ The prostate cancer survival disparity between men with African descent compared with men with European descent is largely explained by access to health care,^2^ but differences in disease presentation at the population level may also contribute.^3^ Yet, genetic ancestry on a global scale has been linked to prostate cancer, with West African ancestry conferring the highest disease risk.^4,5,6,7^ While studies have suggested that modifiable neighborhood factors are causes of cancer disparities,^8,9^ less is known about the interrelationship of West African genetic ancestry and the neighborhood socioeconomic environment in the context of prostate cancer. Previous work by Nagar et al^10^ examining type 2 diabetes reported an interaction between socioeconomic deprivation and genetic ancestry in disease development. Nagar et al^10^ reported that area-level socioeconomic deprivation is a greater risk factor for individuals of African genetic ancestry compared with those with European ancestry.^10^

Racial and ethnic disparities in prostate cancer are the result of complex relationships between both social and environmental neighborhood factors and genetic factors (eg, West African genetic ancestry) across the cancer control continuum.^4,11,12^ In this study, we interpret race as socially constructed by specific context and history, particularly grounded in systemic racism in the US.^13^ The sociopolitical subjugation of people of color in the US has resulted in Black individuals being more likely to reside in segregated neighborhoods with adverse built conditions and limited access to health-promoting resources (eg, healthy foods, quality health care).^14,15,16,17,18^ Although highly correlated with race, we identify West African genetic ancestry as the genetic influences in disease initiation and progression, not associated with racism-related exposures captured by racial categories^13^ and a deconstruction of race.

Currently, genomic risk, including genetic ancestry–inferred risk, is considered a nonmodifiable risk factor. Yet, few studies have examined the role of structural and societal factors in modulating the contribution of genetic susceptibility to prostate cancer risk and outcomes. Understanding how genetic ancestry–inferred risk is modifiable by lived experiences, such as the neighborhood environment in which men at increased risk of adverse prostate cancer outcomes reside, is needed. Examining the intersection of genetic ancestry–inferred risk and racism-related exposures (eg, neighborhood deprivation) may allow for multifactorial tailored interventions that account for both genetic and environmental factors.^10^ Nevertheless, investigations into the influence of neighborhood socioeconomic conditions on the association between West African genetic ancestry and prostate cancer risk remain limited.^19^ Hence, this study investigated whether neighborhood deprivation may modify the association of West African genetic ancestry and prostate cancer risk and mortality.

## Methods

This case-control study was approved by the National Cancer Institute (NCI) and University of Maryland institutional review boards, and this study adhered to the ethical guidelines set by the Declaration of Helsinki. All participants provided written informed consent. This report followed the Strengthening the Reporting of Observational Studies in Epidemiology (STROBE) reporting guideline.

### The NCI-Maryland Prostate Cancer Case-Control Study

Between January 1, 2005, and January 1, 2016, the NCI-Maryland Prostate Cancer Case-Control study enrolled a total of 976 males born in the US (489 self-identified as Black and 487 self-identified as White) diagnosed with prostate cancer. The control group included 1034 (486 Black and 548 White) males. Additional details of the study population and study design are provided in the eMethods in Supplement 1. Individuals with missing baseline home addresses or genetic ancestry data, and individuals with prostate cancer recruited more than 1 year after disease diagnosis were excluded, yielding a final analytic sample of 1469 males: 617 cases (323 Black, 294 White) and 852 controls (410 Black, 442 White). By the end of follow-up, there were 169 all-cause deaths (98 Black men, 71 White men). Excluding 324 males because of missing ancestry data did not significantly change the characteristics of the study population (eTable 1 in Supplement 1).

### Neighborhood Deprivation

Baseline addresses were geocoded using 2010 US Census Tracts Federal Information Processing Series boundaries and were linked to 5-year estimates from the 2000 wave of the American Community Survey (ACS) derived from the National Neighborhood Change Database (Geolytics). The Neighborhood Deprivation Index (NDI) was created based on the approach developed by Messer and colleagues.^20^ Based on prior work that validated the NDI in Maryland,^21^ we used principal components analysis to extract a single factor representing the shared variance of census tract–level socioeconomic standing variables. In the final calculation of the NDI, we retained 6 indicators with loadings greater than 0.25. The following variables are included in the NDI used in this study: percentage of households in poverty, percentage of female-headed households with dependent children, percentage of households on public assistance, percentage of households earning less than $30 000 per year, percentage of males and females unemployed, and percentage of manager occupation. We excluded percentage of high school dropout, percentage owner-occupied crowded housing, and percentage of renter-occupied crowded housing due to not meeting loading criteria of greater than 0.25. The index was standardized to have a mean of 0 and SD of 1, with higher score indicating worse deprivation. Additional details of the NDI creation in this cohort have been described elsewhere.^8^ The NDI was dichotomized at the median (≤median vs >median, in entire cohort) when used in a stratified analysis.

### West African Genetic Ancestry

As previously described,^22^ West African genetic ancestry estimates were obtained from the Cancer Genomics Research Laboratory and NCI-Leidos from a genomewide genotyping study using the Infinium HumanOmni5-Quad BeadChip array. For ancestry estimation, we used a Python-based software approach that uses genomewide single nucleotide variation (SNV) weights precomputed from external reference panels.^23^ We examined a total of 55 446 genotyped SNVs after applying linkage disequilibrium-based pruning and minor allele frequency filtering, and those served as input SNVs. Sensitivity analyses were conducted using a second approach to capture West African genetic ancestry^22^ (eMethods in Supplement 1). We excluded SNVs known to be associated with prostate cancer risk in the 2 approaches to estimate West African genetic ancestry. The population reference panel for the genomewide genotyping approach included Yoruba people from West Africa (113 individuals), Northern European individuals (112 individuals), as well as Han Chinese people and people who identified as having Chinese race and ethnicity living in Denver, Colorado (representing East Asian people; 169 individuals). The population reference panel for the second approach, using 100 ancestry informative markers, included 8 self-reported population groups: participants who identified as White recruited from Washington, District of Columbia, and Chicago, Illinois, represented US people with European ancestry; participants recruited from Sierra Leone (self-reported Mende ethnicity), Nigeria (self-reported Ibo, Hausa, and Yoruba ethnicity) and Cameroon (self-reported as Bamiléké ethnicity), represented West African ancestry; and participants who self-identified as US Cheyenne and Pima represented American Indian ancestry.

### Outcome Variables

Incident prostate cancer was defined as disease diagnosis at least 1 year prior to enrollment. Causes of death were identified through the National Death Index database through December 31, 2020. Patient survival was calculated from date of diagnosis to either date of death or censor date of December 31, 2020. A description of other variables is provided in the eMethods in Supplement 1.

### Statistical Analysis

We assessed the association between West African genetic ancestry and prostate cancer diagnosis using unadjusted and age-adjusted logistic regression to estimate odds ratios (ORs) and 95% CIs. Unadjusted and age-adjusted Cox proportional hazard regression was performed to assess the association between West African genetic ancestry and all-cause mortality to estimate hazard ratios (HRs) and corresponding 95% CIs. In the Cox regression analysis, we additionally included body mass index, smoking, and National Comprehensive Cancer Network risk scores as covariates. All covariates were initially identified a priori. After evaluating each covariate, we excluded covariates that did not alter the estimated main outcome of West African ancestry and neighborhood deprivation by more than 10%. We coded ancestry as a continuous variable, and ORs represent an increase in the estimated odds of prostate cancer per 1 unit of West African genetic ancestry. West African ancestry was coded 0 to 1 (range: 0.04 to 0.84). NDI was also coded as a continuous variable, ranging from −1.37 to 3.93 SDs. We additionally report outcomes representing an increase in the estimated ORs and HRs of prostate cancer diagnosis and mortality per 10% increase in West African genetic ancestry and 1-SD increase in NDI. We calculated 99% CIs in the subanalysis that was restricted to self-identified Black men to report more conservative estimates for this subgroup. In addition, we investigated models for risk and mortality that added NDI. The joint associations of West African genetic ancestry (continuous) and NDI (continuous) with prostate cancer risk and mortality were assessed with multiplicative interaction terms and the likelihood ratio test. We then investigated the association of West African genetic ancestry (continuous) with the odds of a prostate cancer diagnosis and all-cause mortality stratified by NDI (≤median vs >median). We used the Hosmer-Lemeshow goodness-of-fit statistic to evaluate the model fit for logistic regression multivariate models, with *P* > .05 indicating a good model fit. We plotted Cox-Snell residuals compared to the Nelson-Aalen estimator or the cumulative hazard of the residuals to assess fit for mortality models. All analyses were conducted in Stata software version 17.0, and statistical significance was defined as *P* < .05. Analysis was conducted from August 2023 to January 2024.

## Results

Our study included 1469 individuals (mean [SD] age, 64.96 [7.95] years), with 733 Black males and 736 White. Descriptive characteristics are provided in Table 1 and Table 2. West African genetic ancestry was higher among Black cases compared with Black controls (70% vs 67%), and less than 10% of White males had West African ancestry (Table 1). In the total sample, the mean (range) proportion of West African genetic ancestry was 0.27 (0.04-0.84) among participants residing in areas with low NDI and 0.48 (0.07-0.83) among participants residing in areas with high NDI. A greater proportion of Black cases and controls reported not having an education beyond high school and having an income of less than $10 000 per year compared with White cases and controls (Table 1). NDI was highest among Black cases and individuals presenting with regional or metastatic disease (Table 2).

**Table 1.** Descriptive Characteristics of the Total Cohort

Characteristic	Black participants, No. (%)		SMD^a^	White participants, No. (%)		SMD^a^
	Cases (n = 323)	Controls (n = 410)		Cases (n = 294)	Controls (n = 442)	
West African genetic ancestry, mean (SD)	0.70 (0.08)	0.67 (0.09)	−0.28	0.09 (0.02)	0.09 (0.02)	0.09
Age, mean (SD), y	62.87 (7.38)	64.30 (7.81)	0.19	65.37 (7.89)	66.81 (8.09)	0.18
BMI, mean (SD)	28.35 (5.49)	29.55 (5.39)	0.22	28.02 (4.16)	28.09 (5.01)	0.02
Education						
≤High school	160 (49.54)	122 (29.76)	0.60	81 (27.55)	78 (17.65)	0.31
Some college	109 (33.75)	123 (30.00)		78 (26.53)	97 (21.95)	
College	37 (11.46)	85 (20.73)		73 (24.83)	134 (30.32)	
Professional school	16 (4.95)	79 (19.27)		62 (21.09)	133 (30.09)	
Employment status						
Unemployed	220 (68.11)	235 (57.32)	0.22	168 (57.14)	239 (54.07)	0.06
Employed	103 (31.89)	175 (42.68)		126 (42.86)	203 (45.93)	
Income, $						
<10 000	161 (49.85)	89 (21.71)	0.78	68 (23.13)	39 (8.82)	0.42
10 000-30 000	78 (24.15)	86 (20.98)		59 (20.07)	100 (22.62)	
30 000-60 000	31 (9.60)	102 (24.88)		55 (18.71)	106 (23.98)	
60 000-90 000	23 (7.12)	94 (22.93)		93 (31.63)	176 (39.82)	
>90 000	30 (9.29)	39 (9.51)		19 (6.46)	21 (4.75)	

Abbreviations: BMI, body mass index (calculated as weight in kilograms divided by height in meters squared); SMD, standardized mean difference.

^a^ SMD greater than 0.1 indicates meaningful difference.

**Table 2.** Mean Neighborhood Deprivation by Smoking Status and NCCN Risk Score

Neighborhood Deprivation	Black participants, mean (SD)		SMD^a^	White participants, mean (SD)		SMD^a^
	Cases (n = 323)	Controls (n = 410)		Cases (n = 294)	Controls (n = 442)	
Smoking status^b^						
Never	0.79 (1.12)	−0.08 (0.98)	−0.83	−0.54 (0.51)	−0.58 (0.51)	−0.07
Former	0.77 (1.15)	0.02 (0.87)	−0.74	−0.42 (0.57)	−0.50 (0.54)	−0.14
Current	0.94 (.96)	0.79 (1.07)	−0.16	−0.13 (0.83)	−0.27 (0.49)	−0.21
National Comprehensive Cancer Network risk score^c^						
Low	0.69 (1.07)	NA	NA	−0.34 (0.63)	NA	−1.18
Intermediate	0.85 (1.10)	NA	NA	−0.47 (0.63)	NA	−1.47
High or very high	0.80 (1.05)	NA	NA	−0.50 (0.44)	NA	−1.61
Regional or metastatic	1.29 (0.94)	NA	NA	0.02 (0.79)	NA	−1.47

Abbreviations: NA, not applicable; NCCN, National Comprehensive Cancer Network; SMD, standardized mean difference.

^a^ SMD greater than 0.1 indicates meaningful difference.

^b^ SMD between cases and controls by race.

^c^ SMD between Black and White men.

First, we examined the association between West African genetic ancestry and prostate cancer, with and without adjustment for NDI (Table 3 and Figure). In our logistic regression multivariate models, the Hosmer-Lemeshow test supported an overall good model fit, with *P* values ranging from .07 to .63 (Table 3). In the full sample, West African genetic ancestry was positively associated with the odds of prostate cancer (unadjusted OR, 1.49; 95% CI, 1.06-2.11; age-adjusted OR, 1.35; 95% CI, 0.95-1.92). When including West African genetic ancestry and NDI in the same model, West African genetic ancestry was associated with 41% decreased odds of prostate cancer (OR, 0.59; 95% CI, 0.39-0.89), whereas NDI was associated with 70% increased odds of prostate cancer (OR, 1.70; 95% CI, 1.50-1.94). Significant multiplicative interactions were observed between West African genetic ancestry and neighborhood deprivation on risk of a prostate cancer diagnosis (*P* = .02). Analysis stratified by NDI found that West African genetic ancestry was associated with 78% reduced odds of a prostate cancer diagnosis among participants residing in areas with medium to low levels of deprivation (OR, 0.22; 95% CI, 0.11-0.44) (Table 3 and Figure). In contrast, West African genetic ancestry was associated with 98% increased odds of prostate cancer when residing in areas with high levels of neighborhood deprivation (OR, 1.98; 95% CI, 1.23-3.19). Similar patterns were observed in a sensitivity analysis using the West African genetic ancestry estimates derived from 100 ancestry-informative markers (eTable 2 in Supplement 1). When we restricted this analysis to 733 participants who self-identified as Black, no statistically significant interaction was observed (*P* = .07). In the analysis stratified by NDI, West African genetic ancestry was associated with increased odds of prostate cancer when living in neighborhoods with high NDI scores (OR, 18.66; 99% CI, 1.03-337.25; *P* = .009) but not when living in areas with medium to low levels of deprivation (OR, 4.32; 99% CI, 0.04-455.67; *P* = .42) (eTable 3 in Supplement 1).

**Table 3.** Association of West African Ancestry and NDI With a Prostate Cancer Diagnosis and All-Cause Mortality Among Black and White Men

Exposure	Model 1^a^	Model 2^b^	Model 3^c^	NDI-stratified models^d^	
				Low NDI	High NDI
**Prostate cancer diagnosis, OR (95% CI) (n = 1469)** ^e^						
West African ancestry^f^	1.49 (1.06-2.11)^g^	1.35 (0.95-1.92)	0.59 (0.39-0.89)^g^	0.22 (0.11-0.44)^h^	1.98 (1.23-3.19)^i^
Neighborhood Deprivation^f^	1.60 (1.43-1.79)^h^	1.58 (1.41-1.76)^h^	1.70 (1.50-1.94)^h^	NA	NA
*P* value for West African ancestry × Neighborhood Deprivation^j^	NA	NA	.02	NA	NA
**All-cause mortality, HR (95% CI), (n = 616)** ^k^						
West African ancestry^f^	1.68 (1.03-2.75)^g^	1.82 (1.08-3.05)^g^	0.87 (0.46-1.63)	1.18 (0.33-4.14)	1.66 (0.86-3.21)
Neighborhood Deprivation^f^	1.36 (1.20-1.54)^h^	1.42 (1.24-1.62)^h^	1.45 (1.23-1.71)^h^	NA	NA
*P* value for West African ancestry × Neighborhood Deprivation^j^	NA	NA	.44	NA	NA

Abbreviations: HR, hazard ratio; NA, not applicable; NDI, Neighborhood Deprivation Index; OR, odds ratio.

^a^ Model 1 was unadjusted.

^b^ Model 2 adjusted for age at study entry.

^c^ Model 3 adjusted for age at study entry and NDI for the West African ancestry analysis and adjusted for age at study entry and West African ancestry for the NDI analysis.

^d^ NDI was dichotomized at the median (≤median vs >median, using entire cohort). Low deprivation models included 640 participants for the prostate cancer diagnosis analysis and 205 participants for all-cause mortality; high deprivation models included 829 participants for the prostate cancer diagnosis analysis and 411 participants for all-cause mortality.

^e^ Hosmer-Lemeshow test: West African ancestry: model 2, *P* = .27; NDI: model 2, *P* = .07; model 3: *P* = .23; low NDI, *P* = .63; high NDI, *P* = .07.

^f^ West African ancestry estimated using 55 446 genotyped single nucleotide variations and analyzed as continuous.

^g^ *P* < .05.

^h^ *P* < .001.

^i^ *P* < .01.

^j^ Interactions were analyzed in separate models.

^k^ Among cases, there were 169 all-cause events. Mortality models further adjusted for National Comprehensive Cancer Network risk score categories (low, intermediate, high or very high, and regional or metastatic), current body mass index (continuous), and smoking status (never, former, current, missing).

**Figure.**
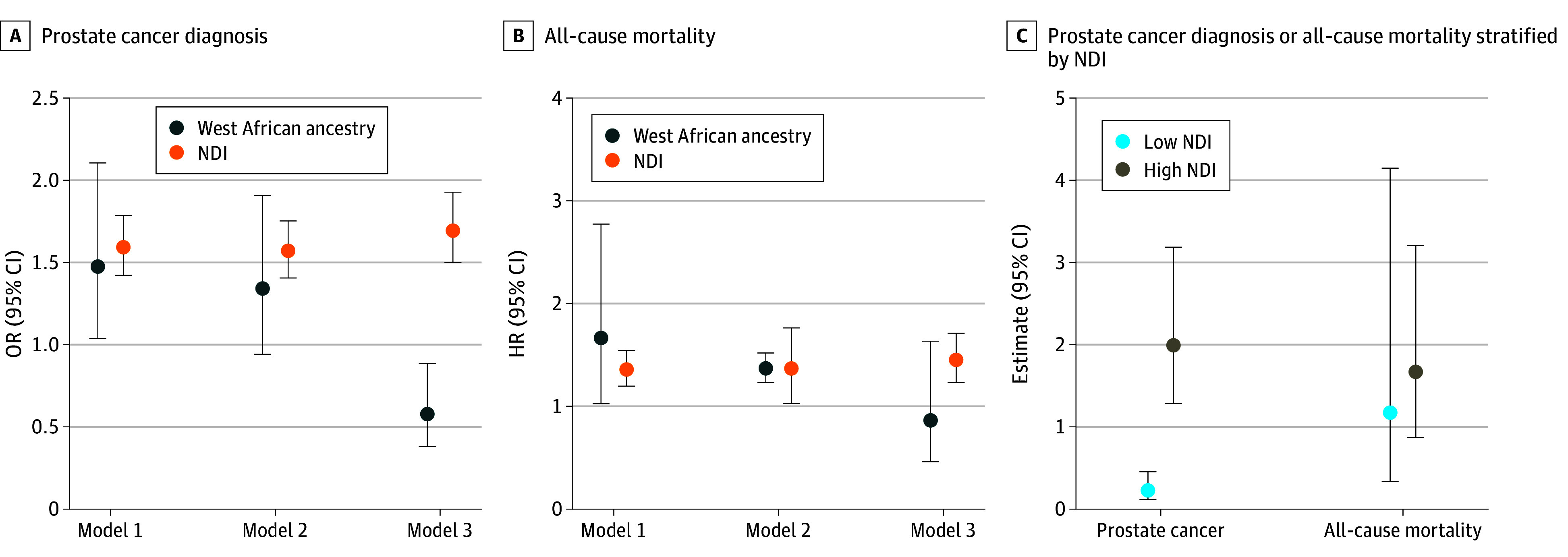
Association of West African Ancestry and Neighborhood Deprivation Index With a Prostate Cancer Diagnosis and All-Cause Mortality A, Odds ratios (OR) and 95% CIs for the association of either West African ancestry or neighborhood deprivation with a prostate cancer diagnosis. B, Hazard ratios (HRs) and 95% CIs for the association of either West African ancestry or neighborhood deprivation with all-cause mortality. Model 1 is unadjusted. Model 2 is age-adjusted. Model 3 adjusts for age and Neighborhood Deprivation Index (NDI) with West African ancestry as the exposure or for age and West African ancestry with NDI as the exposure. Mortality models are additionally adjusted for National Comprehensive Cancer Network risk score categories (low, intermediate, high or very high, and regional or metastatic), current body mass index (continuous), and smoking status (never, former, current, or missing). C, Association of West African ancestry with a prostate cancer diagnosis and all-cause mortality when stratified by NDI. Prostate cancer diagnosis is reported as OR and all-cause mortality as HR. NDI was dichotomized at the median (≤median vs >median). West African ancestry was estimated using 55 446 genotyped single nucleotide variations.

In the mortality analysis, West African genetic ancestry was associated with increased odds of all-cause mortality (unadjusted HR, 1.68; 95% CI, 1.03-2.75; age-adjusted HR, 1.82, 95% CI, 1.08-3.05) (Table 3 and Figure). With further adjustment for NDI, West African genetic ancestry was no longer associated with all-cause mortality. Furthermore, we did not observe a multiplicative interaction between West African genetic ancestry and NDI in our mortality analysis. Similar patterns were observed in sensitivity analysis using West African genetic ancestry estimates based on 100 ancestry-informative markers (eTable 2 and eFigure in Supplement 1).

Lastly, to report ORs and HRs related to defined changes in West African ancestry and NDI, we performed an additional analysis in which we report estimates per 10% increase in West African genetic ancestry and 1 SD increase in NDI for the full sample (eTable 4 in Supplement 1). We found that with every 10% increase in West African genetic ancestry, the risk for prostate cancer increased by an estimated 3% in the age-adjusted analysis, or by 7% when living in a high deprivation area, in the combined cohort.

## Discussion

In this case-control study, we investigated the modifying association of neighborhood-level deprivation on the association of West African genetic ancestry with prostate cancer risk and all-cause mortality. We found that among individuals residing in high-deprivation neighborhoods, West African genetic ancestry was positively associated with increased odds of prostate cancer. In contrast, among individuals residing in more affluent neighborhoods, ancestry was either inversely associated with prostate cancer, indicating a protective association when living in this environment, or did not show an association when restricted to males who identified as Black.

The physical, economic, and social conditions of neighborhoods have been recognized as significant factors associated with cancer outcomes in racially and ethnically diverse populations. Disadvantaged neighborhoods frequently experience higher levels of environmental pollutants, overcrowding, adverse built conditions, and limited access to affordable quality foods and exercise opportunities.^11,12^ Black communities tend to be situated near harmful environmental exposures (eg, major roadways, landfills, industrial sites, Superfund sites) that are associated with an increased risk of cancer.^24,25^ Whereas admixture and genomewide association studies have identified more than 170 common germline risk loci for prostate cancer, including variants at chromosome 8q24, considered to contribute to racial and ethnic differences in prostate cancer risk,^6,26^ there remains scarce evidence that links environmental risk factors to prostate cancer. Nevertheless, some studies indicate a link between air pollution and prostate cancer, as well as worsening environmental quality and odds of metastatic prostate cancer.^27,28,29^ Thus, considering interactions between heritable and environmental factors is important when identifying individuals at high risk of prostate cancer development and mortality.

One mechanism by which the neighborhood environment may exert a biological effect is through increased stress signaling, whereby living in neighborhoods with high levels of deprivation leads to chronic stress and stress-induced proinflammatory signaling and a reduced systemic immune function, as shown in prior studies.^12,30^ Both chronic inflammation and a reduced systemic antitumor immunity are cancer risk factors.^22,31^ Residing in disadvantaged neighborhoods elicits chronic stress, leading to detrimental physiologic responses.^25^ Contextual factors, such as racial profiling, housing discrimination, exposure to violence, and lack of social capital and cohesion, can lead to chronic stress in Black individuals.^25,32^ Excessive stress has been linked to high levels of stress hormones, such as cortisol, which can promote carcinogenesis by augmenting tumor suppressor genes, increasing inflammation and suppressing immunity, and affect tumor microenvironment to support tumor growth, invasion, and metastasis.^32,33,34,35^ Stress signaling, environmental exposures, and altered immune function may then interact with germline genetic risk factors to modulate cancer risk. These interactions may have systemic effects, thereby influencing the process of metastasis, or modify the tumor microenvironment and the biology of a premalignant lesion.

A study by Iyer et al^36^ found that NDI, but not West African genetic ancestry, was associated with all-cause mortality in Black individuals. Iyer et al^36^ concluded that socioeconomic factors were more important determinants of mortality in these men than African genetic ancestry. The findings from our study are consistent with these observations. It is also noteworthy that in most studies that seek to link African genetic ancestry to disease, an adjustment for socioeconomic and lifestyle variables attenuates the association between African ancestry and disease to nonsignificance, eg, studies of cardiovascular disease.^37,38,39^ Our study supports these findings for all-cause mortality among individuals with prostate cancer but found a significant interaction in modifying prostate cancer disease risk for West African genetic ancestry with high neighborhood deprivation, similar to what has been reported for type 2 diabetes in the UK Biobank.^10^ Thus, future research is needed to further investigate this interaction and its underlying biology for prostate cancer development.

### Strengths and Limitations

Our study has several strengths. To our knowledge, this is the first study to report that NDI was significantly associated with modifying the association between West African genetic ancestry and prostate cancer risk. This study undertook a long-term follow-up for all-cause and disease-specific deaths. However, the low number of recorded deaths due to prostate cancer did not permit us to conduct a well-powered interaction analysis for disease-specific mortality. Geocoded addresses at baseline allowed us to construct a reliable and widely used measure of neighborhood deprivation.

Despite these strengths, this study also has some limitations. The cross-sectional design, including collection of addresses at baseline only, limited causal inferences. Specifically, we did not have access to historical addresses and residential mobility or tenure, limiting our ability to capture the associations of early-life and cumulative exposure to neighborhood deprivation. Additionally, the use of census tracts to approximate neighborhoods and recruitment from only the Baltimore, Maryland, region limits generalizability of the findings to other geographic areas. While plausible that genetic and environment interactions vary between population groups,^4^ we were limited in power in detecting variations beyond West African genetic ancestry. Additionally, we were not powered to detect effects among participants presenting with metastatic and lethal disease. Furthermore, our subgroup analysis restricted to Black participants yielded wide CIs and uncertainty in the estimates. Unmeasured confounders, such as health care access (eg, prostate-specific antigen screening participation), might have impacted our results. Additionally, sociocultural factors (eg, nativity, years in the US, heritage) may play a role in these associations; therefore, it is important for future work to explore these associations in other populations. Lastly, individuals may have self-selected into neighborhoods according to individual-level characteristics.

## Conclusions

In this case-control study, we found that neighborhood environments may modify how West African genetic ancestry is associated with prostate cancer risk. Our data indicate that West African genetic ancestry was a greater risk factor for men residing in neighborhoods with high levels of deprivation than for men living in more affluent neighborhoods. While genetic ancestry–inferred risk has been considered a nonmodifiable risk factor, our findings suggest that genetically inferred risk is a modifiable risk factor, not a determination. Thus, improving the neighborhood environment may alleviate both the prostate cancer burden directly and the risk associated with West African genetic ancestry among men residing in areas with high deprivation.

## Notes

Supplement 1.
eMethods.
**eTable 1.** Comparison of Participants With and Without Data on West African Ancestry in the NCI-Maryland Prostate Cancer Case Control Study **eTable 2.** Association of West African Genetic Ancestry (Using 100 AIM) and Neighborhood Deprivation Index With Prostate Cancer Diagnosis and All-Cause Mortality Among Black and White Males **eTable 3.** Association of West African Ancestry and Neighborhood Deprivation Index With a Prostate Cancer Diagnosis Among Black Males **eTable 4.** Association of West African Ancestry (per 10% Increase) and Neighborhood Deprivation Index (per 1 SD Increase) With Prostate Cancer Diagnosis and All-Cause Mortality Among Black and White Males **eFigure.** Association of West African Ancestry (Using 100 AIM) and Neighborhood Deprivation Index With a Prostate Cancer Diagnosis and All-Cause Mortality

Supplement 2.
Data Sharing Statement
