# Error in Abstract and Results

**DOI:** 10.1001/jamanetworkopen.2024.41662

**Published:** 2024-10-14

In the Original Investigation titled “West African Genetic Ancestry, Neighborhood Deprivation, and Prostate Cancer,”^1^ published September 16, 2024, the numbers of self-identified Black and White men were transposed in the Results sections of the Abstract and main text. These should have appeared as 733 Black men and 736 White men. This article has been corrected.^1^
