# Supplementary material for: West African Genetic Ancestry, Neighborhood Deprivation, and Prostate Cancer
Source: JAMA Netw Open. 2024 Sep 16;7(9):e2433546. doi: 10.1001/jamanetworkopen.2024.33546 (PMC11406387; doi:10.1001/jamanetworkopen.2024.33546)
Supplement: Supplement 1. — eMethods. eTable 1. Comparison of Participants With and Without Data on West African Ancestry in the NCI-Maryland Prostate Cancer Case Control Study eTable 2. Association of West African Genetic Ancestry (Using 100 AIM) and Neighborhood Deprivation Index With Prostate Cancer Diagnosis and All-Cause Mortality Among Black and White Males eTable 3. Association of West African Ancestry and Neighborhood Deprivation Index With a Prostate Cancer Diagnosis Among Black Males eTable 4. Association of West African Ancestry (per 10% Increase) and Neighborhood Deprivation Index (per 1 SD Increase) With Prostate Cancer Diagnosis and All-Cause Mortality Among Black and White Males eFigure. Association of West African Ancestry (Using 100 AIM) and Neighborhood Deprivation Index With a Prostate Cancer Diagnosis and All-Cause Mortality [file jamanetwopen-e2433546-s001.pdf]

Supplemental Online Content

Pichardo CM, Ezeani A, Acevedo AM, et al. West African genetic ancestry, neighborhood deprivation, and prostate cancer. *JAMA Netw Open*. 2024;7(9):e2433546. doi:10.1001/jamanetworkopen.2024.33546

eMethods.
eTable 1. Comparison of Participants With and Without Data on West African Ancestry in the NCI-Maryland Prostate Cancer Case Control Study
eTable 2. Association of West African Genetic Ancestry (Using 100 AIM) and Neighborhood Deprivation Index With Prostate Cancer Diagnosis and All-Cause Mortality Among Black and White Males
eTable 3. Association of West African Ancestry and Neighborhood Deprivation Index With a Prostate Cancer Diagnosis Among Black Males
eTable 4. Association of West African Ancestry (per 10% Increase) and Neighborhood Deprivation Index (per 1 SD Increase) With Prostate Cancer Diagnosis and All-Cause Mortality Among Black and White Males
eFigure. Association of West African Ancestry (Using 100 AIM) and Neighborhood Deprivation Index With a Prostate Cancer Diagnosis and All-Cause Mortality

This supplemental material has been provided by the authors to give readers additional information about their work.

© 2024 Pichardo CM et al. *JAMA Network Open.*

eMethods.

Study Population. Between January 1, 2005, and January 1, 2016, the NCI-Maryland Prostate Cancer Case-Control Study enrolled a total of 976 US born males (489 Black and 487 White) diagnosed with prostate cancer within the last 2 years, recruited at the Baltimore Veterans Affairs Medical Center and the University of Maryland Medical Center. Most prostate cancer patients were recruited within 1 year of diagnosis (84%), with a median of 4.80 months between diagnosis and enrollment. The control group included 1034 (486 Black and 548 White) males without a history of cancer other than non-melanoma skin cancer, with a residential working phone number, born in the United States, and fluent in English well enough to be interviewed. Exclusion criteria included a history of radiotherapy or chemotherapy, severe illness, or residing in an institution. Participants self-reported their race and ethnicity. Controls were identified through the Maryland Department of Motor Vehicle database and were frequency-matched by age and race to cases. At time of enrollment, a survey was administered by a trained interviewer, biospecimen were collected, and pathology reports and medical records obtained. Individuals with missing baseline home addresses (n = 86) or genetic ancestry data (n=324), and prevalent cases with recruitment >1 year post disease diagnosis (n = 131), were excluded, yielding a final analytic sample of 1,469 males: 617 cases (323 Black, 294 White) with 169 all-cause (98 Black, 71 White) deaths and 852 controls (410 Black, 442 White). Excluding 324 males because of missing ancestry data did not significantly change the characteristics of the study population (**eTable 1**). The study was approved by the NCI (protocol # 05-C-N021) and University of Maryland (protocol #0298229) Institutional Review Boards. All participants signed an informed consent. In the report, we followed STROBE guidelines for Case-Control studies[1].

Covariates. Individual level socio-demographic indicators and health information were collected utilizing surveys and medical records. We utilized both *a priori* and model building approaches. Our analyses of risk (prostate cancer diagnosis) were controlled for age at study entry. Models of survival were additionally controlled for body mass index (continuous), smoking status (current, former, never, missing) and disease stage defined by National Comprehensive Cancer Network (NCCN) risk scores (low, intermediate, high/very high, regional/metastatic). A description of the classification of cases using NCCN risk scores has been previously published elsewhere [2].

West African genetic ancestry estimation for participants in the NCI-Maryland case-control study. We performed West African genetic ancestry estimation as described previously [2]. Briefly, genomic DNA was isolated from buffy coats (DNeasy Blood & Tissue Kit - Qiagen) or mouthwash samples (standard phenol-chloroform technique). DNA was genotyped by the Cancer Genomics Research Laboratory/NCI-Leidos using the Infinium HumanOmni5-Quad BeadChip array. The resulting large-scale single nucleotide polymorphism (SNP) genotype dataset was then deposited in dbGaP under accession number phs002939.v1.p1 and is publicly available. To estimate West African ancestry from these data, we employed the SNP weights approach for ancestry estimation which is a python-based software for ancestry inference using genome-wide SNP weights precomputed from external reference panels, as previously described [3]**.** We

© 2024 Pichardo CM et al. *JAMA Network Open.*

interrogated a total of 55,446 genotype SNPs after applying linkage disequilibrium-based pruning and minor allele frequency filtering and those served as input SNPs. We used the intersection of SNP weights and input genotypes to perform ancestry inference, and to obtain West African ancestry estimates. The population reference panel used in our analysis included Yoruba people from West Africa (n=113), Northern Europeans (n=112), as well as Han Chinese people and Chinese people living in Denver, US (representing East Asian: ASI; n=169). In a second approach, we genotyped the germline DNA for 100 ancestry informative markers using the Sequenom MassARRAY iPLEX platform, as previously described [4]. Single nucleotide polymorphism (SNP) genotype calls were generated using Sequenom TYPER software. A genotype concordance rate of >99% was observed for all markers. Admixture estimates for each study participant were calculated using a model-based clustering method as implemented in the program STRUCTURE v2.3. We applied STRUCTURE v2.3 with an admixture model estimating K (number of sub populations) from 2 to 5 with 100 iterations and parental population genotypes from West Africans, Europeans, and Native Americans, yielding three admixture estimations (West African, European, Native American). The population reference panel used in this analysis included 8 self-reported population groups: subjects recruited from Washington, DC, and Chicago, IL, representing White/European Americans; subjects recruited from Sierra Leone (self-reported Mende), from Nigeria (self-reported Ibo, Hausa, and Yoruba) and Cameroon (self-reported as Bamileke), representing West African populations; and subjects who self-identified as US Cheyenne and Pima Native American. West African estimates using the different two approaches were similar (r = 0.98) and have been reported by Minas and colleagues [2].

References

1. von Elm E, Altman DG, Egger M, Pocock SJ, Gotzsche PC, Vandenbroucke JP, Initiative S. The Strengthening the Reporting of Observational Studies in Epidemiology (STROBE) statement: guidelines for reporting observational studies. *PLoS Med*. Oct 16 2007;4(10):e296. doi:10.1371/journal.pmed.0040296
2. Minas TZ, Candia J, Dorsey TH, et al. Serum proteomics links suppression of tumor immunity to ancestry and lethal prostate cancer. *Nature Communications*. 2022/04/01 2022;13(1):1759. doi:10.1038/s41467-022-29235-2
3. Chen CY, Pollack S, Hunter DJ, Hirschhorn JN, Kraft P, Price AL. Improved ancestry inference using weights from external reference panels. *Bioinformatics*. Jun 1 2013;29(11):1399-406. doi:10.1093/bioinformatics/btt144
4. Al-Alem U, Rauscher G, Shah E, et al. Association of genetic ancestry with breast cancer in ethnically diverse women from Chicago. *PLoS One*. 2014;9(11):e112916. doi:10.1371/journal.pone.0112916

© 2024 Pichardo CM et al. *JAMA Network Open.*

eTable 1. Comparison of Participants With and Without Data on West African Ancestry in the NCI-Maryland Prostate Cancer Case Control Study

	**Participants with Data on West African Ancestry (N=1,469)**		**Participants with Missing Data on West African Ancestry (N=324)**		**All Participants (N=1,793)**	
	**Cases, n = 617**	**Controls n = 852**	**Cases, n = 153**	**Controls n = 171**	**Cases n = 770**	**Controls n = 1,023**
Age, mean (SD)	64.06 (7.72)	65.60 (8.05)	63.94 (7.95)	66.61 (8.27)	64.04 (7.76)	65.77 (8.09)
Body mass index, mean (SD)	28.19 (4.90)	28.79 (5.25)	28.18 (4.88)	29.93 (5.55)	28.19 (4.90)	28.98 (5.31)
Smoking status, n (%)						
Never	202 (32.74)	341 (40.02)	55 (35.95)	64 (37.43)	257 (33.38)	405 (39.59)
Former	251 (40.68)	380 (44.60)	57 (37.25)	78 (45.61)	308 (40.00)	458 (44.77)
Current	158 (25.61)	122 (14.32)	40 (26.14)	29 (16.96)	198 (25.71)	151 (14.76)
National Comprehensive Cancer Network risk score, n (%)						
Low	118 (19.16)	—	27 (17.65)	—	145 (18.86)	—
Intermediate	322 (52.27)	—	87 (56.86)	—	409 (53.19)	—
High/very high	141 (22.89)	—	34 (22.22)	—	175 (22.76)	—
Regional/metastatic)	35 (5.68)	—	5 (3.27)	—	40 (5.20)	—

© 2024 Pichardo CM et al. *JAMA Network Open.*

eTable 2. Association of West African Genetic Ancestry (Using 100 AIM) and Neighborhood Deprivation Index With Prostate Cancer Diagnosis and All-Cause Mortality Among Black and White Males

				Neighborhood Deprivation Stratified Models [d]	
	Model 1 [a]	Model 2 [b]	Model 3 [c]	Low Neighborhood Deprivation	High Neighborhood Deprivation
**Prostate Cancer Diagnosis OR [e] [95% CI], (n = 1,633)**					
West African ancestry [δ f] (coded as continuous variable)	**1.63*** [1.23, 2.18]**	**1.50** [1.12, 2.01]**	0.76 [0.54, 1.06]	**0.35*** [0.20, 0.62]**	**2.00*** [1.35, 2.97]**
Neighborhood Deprivation[ψ] (coded as continuous variable)	**1.63*** [1.47, 1.81]**	**1.60*** [1.44, 1.78]**	**1.68*** [1.49, 1.89]**	—	—
West African ancestry X Neighborhood Deprivation			*p* = 0.017		
**All-cause mortality, HR [g] [95%CI], (n = 666) [h]**					
West African ancestry [δ f]	**1.61* [1.06, 2.44]**	**1.75* [1.13, 2.72]**	0.99 [0.59, 1.67]	1.71 [0.65, 4.46]	1.55 [0.88, 2.73]
Neighborhood Deprivation [ψ]	**1.35*** [1.19, 1.52]**	**1.40*** [1.23, 1.59]**	**1.40*** [1.20, 1.64]**	—	—
West African ancestry X Neighborhood Deprivation			*p* = 0.224		

Note. Among cases, there were 169 all-causes.

[a] Model 1: unadjusted
[b] Model 2 adjusted for age at study entry
[c] Model 3: [δ]Adjusted for age at study entry and neighborhood deprivation. [ψ]Adjusted for age at study entry and West African ancestry
[d] Neighborhood deprivation index dichotomized at the median (≤ median vs. > median, using entire cohort). Low deprivation models: prostate cancer diagnosis n =703; all-cause mortality n = 219; high deprivation models: prostate cancer diagnosis n = 930; all-cause mortality n = 447
[e] OR: Odds Ratio
[f] West African ancestry estimates were derived from 100 ancestry informative markers (AIM) and analyzed as continuous
[g] HR: Hazard Ratio
[h] Mortality models further adjusted for NCCN risk score categories (low, intermediate, high/very high, regional/metastatic), current body mass index (continuous), and smoking status (never, former, current, missing)

Interactions were analyzed in separate models

* p < 0.05, ** p < 0.01, *** p < 0.001.

© 2024 Pichardo CM et al. *JAMA Network Open.*

eTable 3. Association of West African Ancestry and Neighborhood Deprivation Index With a Prostate Cancer Diagnosis Among Black Males

				Neighborhood Deprivation Stratified Models [d]	
	Model 1 [a]	Model 2 [b]	Model 3 [c]	Low Neighborhood Deprivation	High Neighborhood Deprivation
**Prostate Cancer Diagnosis OR [e] [99% CI], (n = 733)**					
West African ancestry [δ f] (coded as continuous variable)	**27.77*** [2.71, 284.92]**	**24.00*** [2.32, 248.03]**	**10.58* [0.88, 126.83]**	4.32 [0.04, 455.67]	**18.66** [1.03, 337.25]**
Neighborhood Deprivation[ψ] (coded as continuous variable)	**1.90*** [1.56, 2.31]**	**1.89*** [1.55, 2.30]**	**1.85*** [1.52, 2.26]**		
West African ancestry X Neighborhood Deprivation			0.066		

[a] Model 1: unadjusted

[b] Model 2 adjusted for age at study entry

[c] Model 3: [δ]Adjusted for age at study entry and neighborhood deprivation. [ψ]Adjusted for age at study entry and West African ancestry

[d] Neighborhood deprivation index dichotomized at the median (≤ median vs. > median, using entire cohort)

[e] OR: Odds Ratio

[f] West African ancestry estimated using 55,446 genotyped SNPs and analyzed as continuous
Interactions were analyzed in separate models

* p < 0.05, ** p < 0.01, *** p < 0.001

© 2024 Pichardo CM et al. *JAMA Network Open.*

eTable 4. Association of West African Ancestry (per 10% Increase) and Neighborhood Deprivation Index (per 1 SD Increase) With Prostate Cancer Diagnosis and All-Cause Mortality Among Black and White Males

				Neighborhood Deprivation Stratified Models [d]	
	Model 1 [a]	Model 2 [b]	Model 3 [c]	Low Neighborhood Deprivation	High Neighborhood Deprivation
**Prostate Cancer Diagnosis OR [e] [95% CI], (n= 1,469)**					
West African ancestry [δ f] (per 10% increase)	**1.04*** **[1.01, 1.08]**	1.03 [1.00, 1.07]	**0.95*** **[0.91, 0.99]**	**0.86***** **[0.80, 0.92]**	**1.07**** **[1.02, 1.12]**
Neighborhood Deprivation [ψ] (per one SD increase)	**1.60***** **[1.43, 1.79]**	**1.58***** **[1.41, 1.76]**	**1.70***** **[1.50, 1.94]**	—	—
West African ancestry X Neighborhood Deprivation			*p* = 0.024		
**All-cause mortality, HR [g] [95%CI], (n = 616) [h]**					
West African ancestry [δ f]	**1.05*** **[1.00, 1.11]**	**1.06*** **[1.01, 1.12]**	0.99 [0.93, 1.05]	1.02 [0.90, 1.15]	1.05 [0.99, 1.12]
Neighborhood Deprivation [ψ]	**1.36***** **[1.20, 1.54]**	**1.42***** **[1.24,1.62]**	**1.45***** **[1.23, 1.71]**	—	—
West African ancestry X Neighborhood Deprivation			*p* = 0.439		

Note. Among cases, there were 169 all-cause events.

[a] Model 1: unadjusted
[b] Model 2 adjusted for age at study entry
[c] Model 3: [δ]Adjusted for age at study entry and neighborhood deprivation. [ψ]Adjusted for age at study entry and West African ancestry
[d] Neighborhood deprivation index dichotomized at the median (≤ median vs. > median, using entire cohort). Low deprivation models: prostate cancer diagnosis n = 640; all-cause mortality n = 205; high deprivation models: prostate cancer diagnosis n = 829; all-cause mortality n = 411
[e] OR: Odds Ratio
[f] West African ancestry estimated using 55,446 genotyped SNPs and analyzed as continuous.
[g] HR: Hazard Ratio
[h] Mortality models further adjusted for NCCN risk score categories (low, intermediate, high/very high, regional/metastatic), current body mass index (continuous), and smoking status (never, former, current, missing)

Interactions were analyzed in separate models

* $p < 0.05$, ** $p < 0.01$, *** $p < 0.001$.

© 2024 Pichardo CM et al. *JAMA Network Open.*

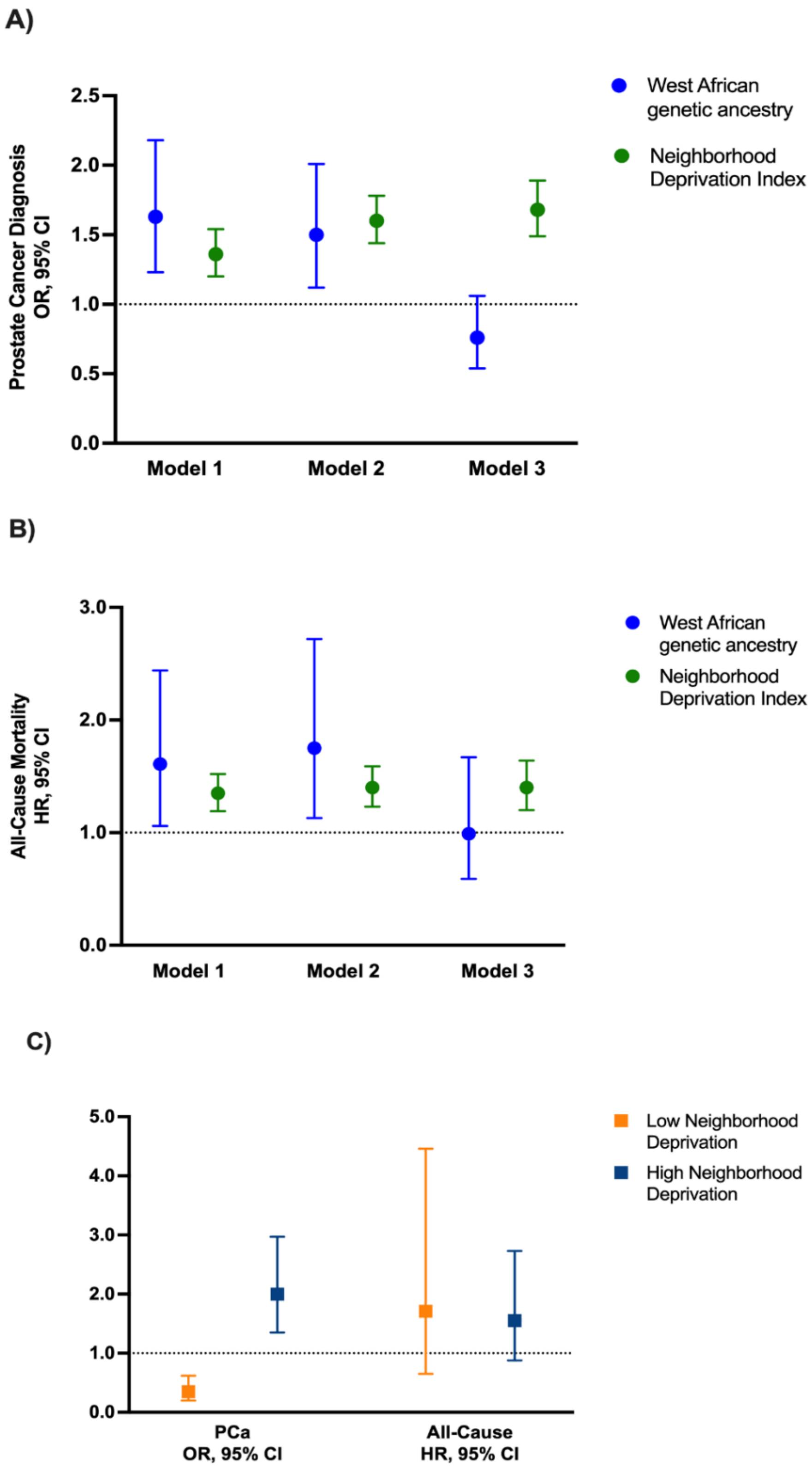

© 2024 Pichardo CM et al. *JAMA Network Open.*

eFigure. Association of West African Ancestry (Using 100 AIM) and Neighborhood Deprivation Index With a Prostate Cancer Diagnosis and All-Cause Mortality

A) Odds ratio (OR) and 95% confidence interval (CI) for association of either West African ancestry or neighborhood deprivation with a prostate cancer diagnosis. B) Hazard ratio (HR) and 95% confidence interval (CI) for their association with all-cause mortality. Model 1 is unadjusted. Model 2 is age-adjusted. Model 3 adjusts for age and neighborhood deprivation with West African ancestry as the exposure or for age and West African ancestry with neighborhood deprivation as the exposure. Mortality models are additionally adjusted for NCCN risk score categories (low, intermediate, high/very high, regional/metastatic), current body mass index (continuous), and smoking status (never, former, current, missing). C) Association of West African ancestry with a prostate cancer diagnosis (PCa) and all-cause mortality (All-Cause) when stratified by neighborhood deprivation index. Odds ratios for a prostate cancer diagnosis and hazard ratios for all-cause mortality. Neighborhood deprivation index was dichotomized at the median (≤ median vs. > median). West African ancestry estimates were derived from 100 ancestry informative markers (AIM).

© 2024 Pichardo CM et al. *JAMA Network Open.*
