# Supplementary material for: West African Genetic Ancestry, Neighborhood Deprivation, and Prostate Cancer
Source: JAMA Netw Open. 2024 Sep 16;7(9):e2433546. doi: 10.1001/jamanetworkopen.2024.33546 (PMC11406387; doi:10.1001/jamanetworkopen.2024.33546)
Supplement: Supplement 2. — Data Sharing Statement [file jamanetwopen-e2433546-s002.pdf]

Data Sharing Statement

Pichardo. West African Genetic Ancestry, Neighborhood Deprivation, and Prostate Cancer. *JAMA Netw Open*. Published September 16, 2024. doi:10.1001/jamanetworkopen.2024.33546

Data
Data available: Yes
Data types: Deidentified participant data
How to access data: https://doi.org/10.17605/OSF.IO/327HA ; https://doi.org/10.5281/zenodo.5815262
When available: With publication

Supporting Documents
Document types: None

Additional Information
Who can access the data: anyone requesting the data
Types of analyses: Statistical/analytic code
Mechanisms of data availability: with investigator support
Any additional restrictions: no restrictions; anybody who is asking for the data will receive those
